# Supplementary material for: Heparin-based hydrogel scaffolding alters the transcriptomic profile and increases the chemoresistance of MDA-MB-231 triple-negative breast cancer cells
Source: Biomater Sci. 2020 Feb 13;8(10):2786–96. doi: 10.1039/c9bm01481k (PMC7497406; doi:10.1039/c9bm01481k)
Supplement: Supplementary file 2 [file BM-008-C9BM01481K-s002.zip › Supplementary File 4/HP-BhydrogelvControl/Pathways/my_analysis.Gsea.1545201079907/HALLMARK_ESTROGEN_RESPONSE_LATE.html]

Details for gene set HALLMARK\_ESTROGEN\_RESPONSE\_LATE[GSEA]

|  || Dataset | expr.class.cls#HYD\_versus\_CONTROL.class.cls#HYD\_versus\_CONTROL\_repos |
| Phenotype | class.cls#HYD\_versus\_CONTROL\_repos |
| Upregulated in class | CONTROL |
| GeneSet | HALLMARK\_ESTROGEN\_RESPONSE\_LATE |
| Enrichment Score (ES) | -0.26719987 |
| Normalized Enrichment Score (NES) | -1.2345704 |
| Nominal p-value | 0.054945055 |
| FDR q-value | 0.12049074 |
| FWER p-Value | 0.685 |
Table: GSEA Results Summary

  

Fig 1: Enrichment plot: HALLMARK\_ESTROGEN\_RESPONSE\_LATE      
 Profile of the Running ES Score & Positions of GeneSet Members on the Rank Ordered List

  

| PROBE | DESCRIPTION (from dataset) | GENE SYMBOL | GENE\_TITLE | RANK IN GENE LIST | RANK METRIC SCORE | RUNNING ES | CORE ENRICHMENT || 1 | CACNA2D2 | na |  |  | 323 | 2.634 | 0.0055 | No |
| 2 | XRCC3 | na |  |  | 419 | 2.448 | 0.0214 | No |
| 3 | HOMER2 | na |  |  | 502 | 2.341 | 0.0371 | No |
| 4 | PTPN6 | na |  |  | 544 | 2.288 | 0.0545 | No |
| 5 | SLC22A5 | na |  |  | 826 | 2.011 | 0.0569 | No |
| 6 | DLG5 | na |  |  | 1017 | 1.862 | 0.0628 | No |
| 7 | LLGL2 | na |  |  | 1042 | 1.843 | 0.0773 | No |
| 8 | SLC2A8 | na |  |  | 1116 | 1.788 | 0.0887 | No |
| 9 | RAPGEFL1 | na |  |  | 1274 | 1.703 | 0.0950 | No |
| 10 | ID2 | na |  |  | 1383 | 1.637 | 0.1033 | No |
| 11 | NCOR2 | na |  |  | 1743 | 1.474 | 0.0970 | No |
| 12 | PKP3 | na |  |  | 2554 | 1.158 | 0.0644 | No |
| 13 | BAG1 | na |  |  | 2573 | 1.152 | 0.0733 | No |
| 14 | SCARB1 | na |  |  | 2693 | 1.119 | 0.0766 | No |
| 15 | MAPK13 | na |  |  | 2746 | 1.104 | 0.0832 | No |
| 16 | TSTA3 | na |  |  | 2802 | 1.086 | 0.0896 | No |
| 17 | ST14 | na |  |  | 3433 | 0.931 | 0.0645 | No |
| 18 | DNAJC1 | na |  |  | 3493 | 0.920 | 0.0692 | No |
| 19 | CYP26B1 | na |  |  | 3501 | 0.917 | 0.0767 | No |
| 20 | FOXC1 | na |  |  | 3523 | 0.912 | 0.0834 | No |
| 21 | ITPK1 | na |  |  | 3751 | 0.858 | 0.0788 | No |
| 22 | SEMA3B | na |  |  | 3816 | 0.847 | 0.0827 | No |
| 23 | PLXNB1 | na |  |  | 4368 | 0.738 | 0.0600 | No |
| 24 | CELSR2 | na |  |  | 4443 | 0.725 | 0.0623 | No |
| 25 | TPBG | na |  |  | 4814 | 0.656 | 0.0485 | No |
| 26 | TH | na |  |  | 4938 | 0.636 | 0.0474 | No |
| 27 | FAM102A | na |  |  | 5184 | 0.602 | 0.0397 | No |
| 28 | SLC7A5 | na |  |  | 5187 | 0.602 | 0.0447 | No |
| 29 | FARP1 | na |  |  | 5271 | 0.589 | 0.0454 | No |
| 30 | IGSF1 | na |  |  | 5544 | 0.545 | 0.0358 | No |
| 31 | CDH1 | na |  |  | 5848 | 0.507 | 0.0242 | No |
| 32 | FGFR3 | na |  |  | 5871 | 0.504 | 0.0273 | No |
| 33 | EGR3 | na |  |  | 6013 | 0.483 | 0.0241 | No |
| 34 | TPD52L1 | na |  |  | 6047 | 0.480 | 0.0264 | No |
| 35 | ALDH3B1 | na |  |  | 6050 | 0.480 | 0.0304 | No |
| 36 | TFAP2C | na |  |  | 6126 | 0.467 | 0.0305 | No |
| 37 | FOS | na |  |  | 6167 | 0.461 | 0.0323 | No |
| 38 | METTL3 | na |  |  | 6245 | 0.452 | 0.0321 | No |
| 39 | MAPT | na |  |  | 6363 | 0.435 | 0.0297 | No |
| 40 | IL17RB | na |  |  | 6523 | 0.413 | 0.0249 | No |
| 41 | TJP3 | na |  |  | 6670 | 0.396 | 0.0206 | No |
| 42 | DCXR | na |  |  | 6794 | 0.384 | 0.0174 | No |
| 43 | SLC1A4 | na |  |  | 6805 | 0.382 | 0.0201 | No |
| 44 | SLC9A3R1 | na |  |  | 6845 | 0.378 | 0.0213 | No |
| 45 | LSR | na |  |  | 6876 | 0.374 | 0.0229 | No |
| 46 | NAB2 | na |  |  | 6881 | 0.373 | 0.0259 | No |
| 47 | AMFR | na |  |  | 6900 | 0.371 | 0.0281 | No |
| 48 | PDZK1 | na |  |  | 7086 | 0.350 | 0.0214 | No |
| 49 | KLF4 | na |  |  | 7185 | 0.341 | 0.0191 | No |
| 50 | CCND1 | na |  |  | 7494 | 0.308 | 0.0056 | No |
| 51 | AFF1 | na |  |  | 7495 | 0.308 | 0.0082 | No |
| 52 | ABCA3 | na |  |  | 7496 | 0.308 | 0.0109 | No |
| 53 | JAK2 | na |  |  | 7561 | 0.298 | 0.0100 | No |
| 54 | BCL2 | na |  |  | 7623 | 0.289 | 0.0093 | No |
| 55 | MICB | na |  |  | 7630 | 0.288 | 0.0115 | No |
| 56 | RPS6KA2 | na |  |  | 7768 | 0.277 | 0.0066 | No |
| 57 | ANXA9 | na |  |  | 7794 | 0.273 | 0.0076 | No |
| 58 | SNX10 | na |  |  | 7849 | 0.267 | 0.0071 | No |
| 59 | DHRS2 | na |  |  | 7852 | 0.267 | 0.0093 | No |
| 60 | ETFB | na |  |  | 8173 | 0.236 | -0.0055 | No |
| 61 | CHPT1 | na |  |  | 8205 | 0.231 | -0.0052 | No |
| 62 | WFS1 | na |  |  | 8337 | 0.215 | -0.0102 | No |
| 63 | CDC6 | na |  |  | 8605 | 0.191 | -0.0226 | No |
| 64 | PRLR | na |  |  | 8658 | 0.185 | -0.0238 | No |
| 65 | UNC13B | na |  |  | 8672 | 0.183 | -0.0229 | No |
| 66 | ASS1 | na |  |  | 8674 | 0.183 | -0.0214 | No |
| 67 | SGK1 | na |  |  | 9100 | 0.141 | -0.0425 | No |
| 68 | PPIF | na |  |  | 9104 | 0.140 | -0.0415 | No |
| 69 | CKB | na |  |  | 9267 | 0.121 | -0.0490 | No |
| 70 | GINS2 | na |  |  | 9453 | 0.096 | -0.0578 | No |
| 71 | CDC20 | na |  |  | 9617 | 0.086 | -0.0657 | No |
| 72 | XBP1 | na |  |  | 9718 | 0.073 | -0.0703 | No |
| 73 | PRKAR2B | na |  |  | 9760 | 0.070 | -0.0719 | No |
| 74 | SERPINA5 | na |  |  | 9913 | 0.052 | -0.0794 | No |
| 75 | FKBP5 | na |  |  | 9936 | 0.048 | -0.0801 | No |
| 76 | DHCR7 | na |  |  | 10032 | 0.036 | -0.0848 | No |
| 77 | MYB | na |  |  | 10213 | 0.015 | -0.0942 | No |
| 78 | BATF | na |  |  | 10429 | 0.004 | -0.1054 | No |
| 79 | SCNN1A | na |  |  | 10551 | -0.010 | -0.1117 | No |
| 80 | CCNA1 | na |  |  | 10576 | -0.014 | -0.1128 | No |
| 81 | ZFP36 | na |  |  | 10619 | -0.019 | -0.1149 | No |
| 82 | TMPRSS3 | na |  |  | 10856 | -0.046 | -0.1269 | No |
| 83 | PDLIM3 | na |  |  | 10867 | -0.049 | -0.1270 | No |
| 84 | GALE | na |  |  | 10999 | -0.065 | -0.1333 | No |
| 85 | CD44 | na |  |  | 11081 | -0.075 | -0.1369 | No |
| 86 | FKBP4 | na |  |  | 11149 | -0.086 | -0.1397 | No |
| 87 | SIAH2 | na |  |  | 11609 | -0.127 | -0.1627 | No |
| 88 | PTGES | na |  |  | 11658 | -0.133 | -0.1641 | No |
| 89 | ALDH3A2 | na |  |  | 11689 | -0.137 | -0.1645 | No |
| 90 | MYOF | na |  |  | 11838 | -0.156 | -0.1710 | No |
| 91 | NXT1 | na |  |  | 11839 | -0.156 | -0.1696 | No |
| 92 | LAMC2 | na |  |  | 11847 | -0.157 | -0.1687 | No |
| 93 | CLIC3 | na |  |  | 11878 | -0.160 | -0.1689 | No |
| 94 | SFN | na |  |  | 12185 | -0.198 | -0.1833 | No |
| 95 | ATP2B4 | na |  |  | 12320 | -0.216 | -0.1885 | No |
| 96 | MEST | na |  |  | 12346 | -0.220 | -0.1879 | No |
| 97 | RBBP8 | na |  |  | 12693 | -0.242 | -0.2040 | No |
| 98 | RABEP1 | na |  |  | 12748 | -0.252 | -0.2047 | No |
| 99 | FABP5 | na |  |  | 13043 | -0.289 | -0.2177 | No |
| 100 | NBL1 | na |  |  | 13075 | -0.295 | -0.2168 | No |
| 101 | FRK | na |  |  | 13207 | -0.312 | -0.2210 | No |
| 102 | TNNC1 | na |  |  | 13325 | -0.327 | -0.2243 | No |
| 103 | ISG20 | na |  |  | 13723 | -0.391 | -0.2419 | No |
| 104 | PLK4 | na |  |  | 13826 | -0.410 | -0.2437 | No |
| 105 | SERPINA1 | na |  |  | 14126 | -0.430 | -0.2557 | No |
| 106 | ABHD2 | na |  |  | 14131 | -0.431 | -0.2523 | No |
| 107 | MOCS2 | na |  |  | 14212 | -0.444 | -0.2527 | No |
| 108 | TST | na |  |  | 14230 | -0.448 | -0.2498 | No |
| 109 | STIL | na |  |  | 14238 | -0.449 | -0.2463 | No |
| 110 | IDH2 | na |  |  | 14279 | -0.459 | -0.2445 | No |
| 111 | FDFT1 | na |  |  | 14374 | -0.474 | -0.2454 | No |
| 112 | HSPA4L | na |  |  | 14452 | -0.483 | -0.2453 | No |
| 113 | IMPA2 | na |  |  | 14700 | -0.523 | -0.2538 | No |
| 114 | MDK | na |  |  | 14716 | -0.526 | -0.2501 | No |
| 115 | CPE | na |  |  | 14839 | -0.543 | -0.2519 | No |
| 116 | TIAM1 | na |  |  | 14991 | -0.561 | -0.2550 | No |
| 117 | WISP2 | na |  |  | 15043 | -0.569 | -0.2529 | No |
| 118 | TRIM29 | na |  |  | 15120 | -0.584 | -0.2519 | No |
| 119 | ST6GALNAC2 | na |  |  | 15320 | -0.621 | -0.2570 | No |
| 120 | ARL3 | na |  |  | 15415 | -0.636 | -0.2565 | No |
| 121 | ACOX2 | na |  |  | 15508 | -0.652 | -0.2558 | No |
| 122 | BLVRB | na |  |  | 15546 | -0.661 | -0.2521 | No |
| 123 | CISH | na |  |  | 15622 | -0.672 | -0.2503 | No |
| 124 | PAPSS2 | na |  |  | 15945 | -0.732 | -0.2610 | Yes |
| 125 | TOP2A | na |  |  | 15987 | -0.741 | -0.2568 | Yes |
| 126 | CA12 | na |  |  | 16047 | -0.759 | -0.2534 | Yes |
| 127 | SLC29A1 | na |  |  | 16048 | -0.759 | -0.2469 | Yes |
| 128 | JAK1 | na |  |  | 16113 | -0.771 | -0.2437 | Yes |
| 129 | OPN3 | na |  |  | 16178 | -0.783 | -0.2404 | Yes |
| 130 | TSPAN13 | na |  |  | 16213 | -0.790 | -0.2354 | Yes |
| 131 | CAV1 | na |  |  | 16368 | -0.825 | -0.2365 | Yes |
| 132 | TFPI2 | na |  |  | 16544 | -0.865 | -0.2383 | Yes |
| 133 | GLA | na |  |  | 16858 | -0.944 | -0.2466 | Yes |
| 134 | EMP2 | na |  |  | 16921 | -0.962 | -0.2417 | Yes |
| 135 | KRT19 | na |  |  | 16930 | -0.965 | -0.2339 | Yes |
| 136 | AGR2 | na |  |  | 16965 | -0.974 | -0.2273 | Yes |
| 137 | BTG3 | na |  |  | 16989 | -0.983 | -0.2202 | Yes |
| 138 | SLC26A2 | na |  |  | 17094 | -1.013 | -0.2170 | Yes |
| 139 | RNASEH2A | na |  |  | 17184 | -1.044 | -0.2127 | Yes |
| 140 | KIF20A | na |  |  | 17258 | -1.069 | -0.2075 | Yes |
| 141 | ADD3 | na |  |  | 17325 | -1.093 | -0.2016 | Yes |
| 142 | NMU | na |  |  | 17376 | -1.112 | -0.1947 | Yes |
| 143 | TFF1 | na |  |  | 17389 | -1.117 | -0.1858 | Yes |
| 144 | HR | na |  |  | 17401 | -1.118 | -0.1769 | Yes |
| 145 | RAB31 | na |  |  | 17417 | -1.120 | -0.1681 | Yes |
| 146 | PLA2G16 | na |  |  | 17571 | -1.185 | -0.1660 | Yes |
| 147 | TOB1 | na |  |  | 17952 | -1.348 | -0.1745 | Yes |
| 148 | AREG | na |  |  | 18122 | -1.456 | -0.1709 | Yes |
| 149 | COX6C | na |  |  | 18290 | -1.558 | -0.1664 | Yes |
| 150 | NRIP1 | na |  |  | 18335 | -1.591 | -0.1551 | Yes |
| 151 | UGDH | na |  |  | 18399 | -1.641 | -0.1444 | Yes |
| 152 | ELOVL5 | na |  |  | 18504 | -1.707 | -0.1353 | Yes |
| 153 | PDCD4 | na |  |  | 18523 | -1.724 | -0.1215 | Yes |
| 154 | IL6ST | na |  |  | 18809 | -2.032 | -0.1192 | Yes |
| 155 | PRSS23 | na |  |  | 18840 | -2.072 | -0.1030 | Yes |
| 156 | CD9 | na |  |  | 18879 | -2.138 | -0.0868 | Yes |
| 157 | HPRT1 | na |  |  | 18898 | -2.164 | -0.0693 | Yes |
| 158 | PERP | na |  |  | 18900 | -2.167 | -0.0508 | Yes |
| 159 | SORD | na |  |  | 18930 | -2.228 | -0.0333 | Yes |
| 160 | DYNLT3 | na |  |  | 19045 | -2.502 | -0.0179 | Yes |
| 161 | HSPB8 | na |  |  | 19137 | -3.038 | 0.0032 | Yes |
Table: GSEA details [plain text format]

  

Fig 2: HALLMARK\_ESTROGEN\_RESPONSE\_LATE      
 Blue-Pink O' Gram in the Space of the Analyzed GeneSet

  

Fig 3: HALLMARK\_ESTROGEN\_RESPONSE\_LATE: Random ES distribution      
 Gene set null distribution of ES for **HALLMARK\_ESTROGEN\_RESPONSE\_LATE**
